# A genomic predictor of lifespan in vertebrates

**DOI:** 10.1038/s41598-019-54447-w

**Published:** 2019-12-12

**Authors:** Benjamin Mayne, Oliver Berry, Campbell Davies, Jessica Farley, Simon Jarman

**Affiliations:** 1 Environomics Future Science Platform, Indian Oceans Marine Research Centre, Commonwealth Scientific and Industrial Research Organisation, Crawley, Western Australia Australia; 2 Oceans and Atmosphere, Commonwealth Scientific and Industrial Research Organisation, Hobart, Tasmania Australia; 3 School of Biological Sciences, University of Western Australia, 35 Stirling Highway, Perth, Western Australia Australia

**Keywords:** Ecological genetics, Conservation genomics

## Abstract

Biological ageing and its mechanistic underpinnings are of immense biomedical and ecological significance. Ageing involves the decline of diverse biological functions and places a limit on a species’ maximum lifespan. Ageing is associated with epigenetic changes involving DNA methylation. Furthermore, an analysis of mammals showed that the density of CpG sites in gene promoters, which are targets for DNA methylation, is correlated with lifespan. Using 252 whole genomes and databases of animal age and promotor sequences, we show a pattern across vertebrates. We also derive a predictive lifespan clock based on CpG density in a selected set of promoters. The lifespan clock accurately predicts maximum lifespan in vertebrates (R^2^ = 0.76) from the density of CpG sites within only 42 selected promoters. Our lifespan clock provides a wholly new method for accurately estimating lifespan using genome sequences alone and enables estimation of this challenging parameter for both poorly understood and extinct species.

## Introduction

Biological ageing is observed in almost all animal species^1,2^. Ageing involves the decline of diverse biological functions and the dynamics of this process limit a species’ maximum lifespan^3^. Longevity of individuals is strongly linked to specific alleles in genetic model organisms^4–6^. Ageing is also associated with several epigenetic changes involving DNA methylation (DNAm)^7,8^. DNAm of cytosine-phosphate-guanosine (CpG) sites, involves a covalent modification to cytosine to form 5-methylcytosine. This modification to DNA has the potential to regulate gene expression, including of genes critical for longevity, without altering the underlying sequence. The observation that DNAm at promotor CpG sites can accumulate or decline predictably with age, over and above the more random process of epigenetic drift [19], has enabled the development of “clock like” biomarkers for age^9–11^. Individual human age, for example, can be predicted with great accuracy (R^2^ = 0.92) in a range of tissues by an epigenetic clock^12^. Similar epigenetic clocks have been created in a range of mammal and bird species^13–17^.

Although it’s often reported, maximum lifespan for a species is difficult to define. It is frequently the highest reported value for captive animals because of the difficulty in estimating age for wild individuals. Alternatively, it is an accepted consensus value for the majority of individuals within a species^18^, or is based on records from a small number of wild individuals that have an age estimate as a result of exceptional circumstances^14,19^. Maximum lifespans differ greatly among species, even among fairly closely-related species^20^. In vertebrates, species such as the pygmy goby (*Eviota sigillata*) live for only eight weeks^21^, while the Greenland shark (*Somniosus microcephalus*) may live for more than 400 years^22^. In mammals, the forest shrew (*Myosorex varius*) has one of the shortest reported lifespans at 2.1 years^23^, whereas some bowhead whales (*Balaena mysticeta*) have been reported to be older than 200 years^19,23^. The differences in lifespan between species has ecological significance because age regulates fundamental aspects of animal life cycles and demography such as probability of mortality^24^. Consequently lifespan is central to estimating risk of animal extinction^25^, evaluating biosecurity risks^26^ and estimating the sustainable yield in fisheries and other harvested organisms^27^. Yet, despite this profound practical importance, lifespan is poorly characterised for most wild animals because it is difficult to estimate^28^.

Maximum lifespan is believed to be under genetic control^23–25^, but so far, no gene variants can account for differences in lifespan among species. Because ageing is characterised by changes in gene expression caused by DNAm, another potential controller of lifespan is genomic changes that accommodate DNAm’s effects on regulation of gene expression. Specifically, clusters of high density CpG sites, also known as CpG islands, are highly conserved within promoter sequences^29,30^ and well known for regulating gene expression^31^. CpG sites are also prone to mutation^32^ and their function in regulating gene expression may make them prime targets for evolutionary pressures to vary lifespans.

This hypothesis was strongly supported in an investigation showing that CpG density is correlated with lifespan among a set of conserved mammalian promoters^26^. McLain and Faulk^33^ identified 1,079 promoters where the CpG density correlated with increasing lifespan (q < 0.05). This suggests a functional role for CpG density in the maximum lifespan of mammalian species. Highly dense CpG regions may offer greater buffering to long-lived species against dysregulation caused by accumulated methylation^33^. Despite promoters being conserved across vertebrates^34^, it is unknown if CpG density within promoters are a key driver of the expression of genes related to lifespan. Moreover, although many studies have explored using the predictive power of methylation at specific CpG sites, no such study has investigated the predictive power of CpG density to estimate lifespan.

Here, we extend observations of the correlation between promoter CpG density and lifespan in mammals to produce a predictive model for lifespan in all vertebrates. We use reference genomes of animals with known lifespans to identify promoters that can be predictive of lifespan. We combined data from major databases including NCBI Genomes^35^, the Eukaryotic Promoter Database (EPD)^36^, Animal Ageing and Longevity Database (AnAge)^23^ and TimeTree^37^ to build a predictive model that estimates lifespan. Our results show CpG density in selected promoters is highly predictive of lifespan across vertebrates. To our knowledge this is the first study which has built a genetic predictive model to estimate the lifespan of vertebrate species from genetic markers.

## Results

### Lifespan estimation from CpG density

We identified all vertebrate species that had reference genomes available in NCBI^35^, known maximum lifespans in the AnAge database^23^ and evolutionary divergence times in TimeTree^37^. This primary data set contained 252 species from five vertebrate classes (Supplementary Table 1), with lifespans ranging from 1.1 years, a Turquoise killifish (*Nothobranchius furzeri*) to 205 years, a Rougheye rockfish (*Sebastes aleutianus*). We removed humans (*Homo sapiens*) from the data set as they were listed with a maximum lifespan of 120 years, which does not reflect the variability and the true global average lifespan (60.9–86.3 years)^38^.

Mammals comprised the most represented class of vertebrates in the data set (Supplementary Table 1), and the average BLAST length of promoters from EPD was 374 bp (Supplementary Fig. 2). The BLAST hit length decreased with increasing evolutionary distance from humans (R^2^ = −0.85, p-value < 2.20 × 10^−16^; Supplementary Fig. 2), which is most likely a reflection of using human promoter sequences. We also identified a positive correlation (R^2^ = 0.64, p-value < 2.2 × 10^−16^) between total CpG sites and genome size across animal species (Supplementary Fig. 3). It has been suggested that an increase rate of recombination prevents the loss of CpG island density during increased chromosome numbers and genome size^39^. This suggests that CpG density genome wide is maintained across different sized animal genomes.

**Figure 1:**
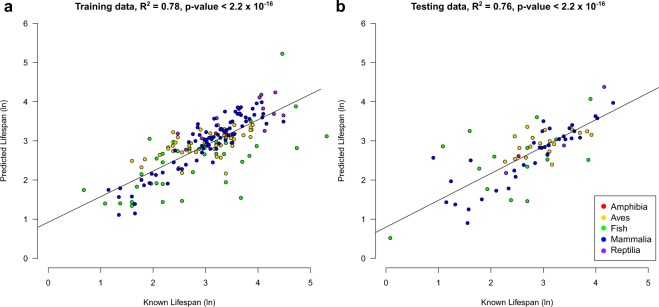
Lifespan Estimation from CpG density with lifespan loci. The correlation between the known and predicted lifespan in the (**a**) training and (**b**) testing data set. Colours denote the class of each species. The R^2^ value and p-value are given above each plot.

The final lifespan predictor was based on 42 promoters (Supplementary Table 2) after a 10-fold cross validation to optimise the model (see Methods). From here on, promoters in the model will be referred to as the lifespan loci and the model itself as the lifespan clock. As expected, the lifespan clock returned a regression coefficient between the known and predicted lifespan of species within the training data set (R^2^ = 0.78, p-value < 2.20 × 10^−16^) (Fig. 1a). Furthermore, using the independent set of samples in the testing data set, the lifespan clock also returned a high regression coefficient (R^2^ = 0.76, p-value < 2.20 × 10^−16^; Fig. 1b). In addition, the correlation between the known and predicted lifespan using untransformed log values was 0.77 and 0.76 for the training and testing data set respectively. Although the model was developed using all classes of vertebrates, which was accounted for using a phylogenetic generalized least squares (PGLS) approach (see methods), it is important to note that multiple slopes of regression may exist in classes of vertebrates. Therefore, more tailored models may be potentially developed specific to a species class or taxonomy. This was confirmed using an ANCOVA test which showed a significant effect (p-value = 0.00014) of vertebrate class with predicted maximum lifespan. Moreover, in the testing data set using untransformed log values of lifespan we found statistically significant but differing regression coefficients for each vertebrate class between the known and predicted lifespans (Aves; R^2^ = 0.49, p-value = 0.043, Fish; R^2^ = 0.56, p-value = 0.025, Mammalia; R^2^ = 0.91, p-value = 1.85 × 10^−13^, Reptilia; R^2^ = 0.94, p-value = 0.029). We were unable to dwetermine the regression coefficient for Amphibia due to low sample size in the testing data set. Other lifespan prediction models could potentially be developed in the future, specific to species class or taxonomic ranking as the AnAge database continues to gain more lifespan data and more reference genomes become available. The accuracy in predicting the lifespan of species from all five vertebrate classes examined (Supplementary Table 1 and Fig. 1b), suggests that CpG density has the application of a universal bio-marker panel for lifespan in vertebrates.

**Figure 2:**
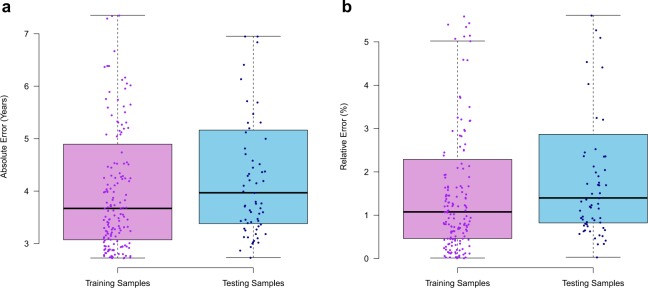
Performance and characterisation of the lifespan loci. Box plots show the (**a**) Absolute error rate, (**b**) relative error rate of each species in the training and testing data sets. Each dot point overlayed on the box plots represent an individual species.

**Figure 3:**
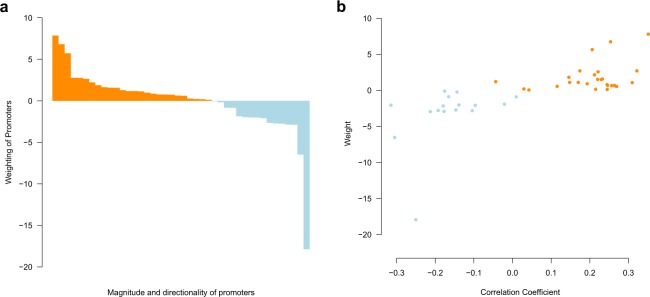
Weighting and correlation coefficients of the lifespan loci. (**a**) Weighting of each lifespan loci in order from most positive to negative in magnitude. (**b**) Pearson correlation compared to the weight of each lifespan loci.

**Figure 4:**
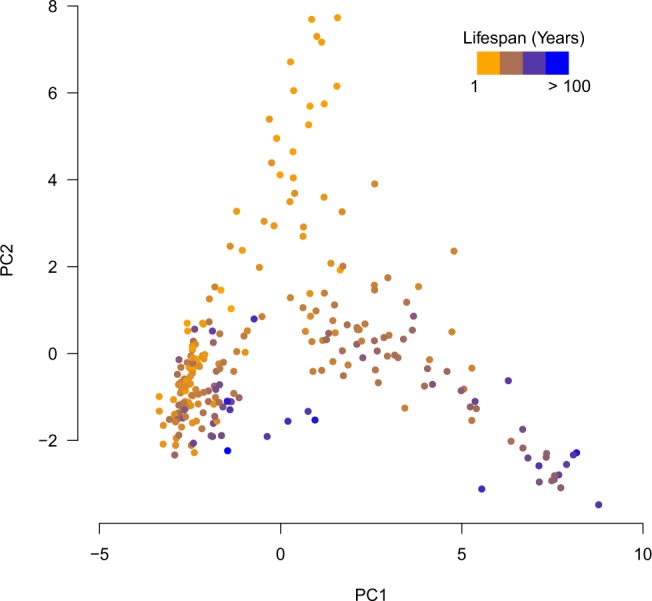
Principle component analysis using the CpG density in the lifespan loci which shows the species separate based on their known lifespans. Species are coloured by increasing lifespan.

The lifespan clock performed well across species from all classes, producing a median absolute error (MAE) of 3.72 years (Fig. 2a) and a maximum relative error of 5.9% (Fig. 2b) in the testing data set. We also found no significant difference between the absolute error rate between the training and testing data sets (p = 0.20, t-test). In the testing dataset no difference between MAE was found between species that had their lifespan estimates obtained from either captivity (43 species) or the wild (26 species) (p = 0.31, t-test). This suggests that the source of the lifespan estimate from the AnAge database (captivity or wild) was not a major confounding factor to the model. Despite high accuracy, individual lifespan loci may not necessarily represent the strongest lifespan correlated promoters (Fig. 3a,b). This is similar to other age-related models, where individual components of the overall model do not necessarily correlate well with the age-related feature^13,40^. Therefore, the lifespan loci may only be somewhat predictive of the directionality of CpG density with increasing lifespan. Principle component analysis (PCA) was used to visually characterise the variation of CpG density in the different species. A PCA of the lifespan loci will elucidate the extent to which the species separate out by lifespan and if there are other drivers of variation in CpG density within the species. The PCA separated the species based on lifespan (Fig. 4). This analysis suggests the CpG density of the lifespan loci separate species based on lifespan well. It also suggests technical variations such as the genome assembly level, (e.g. contig, scaffold, chromosome assembly) are not a major source of variation between samples (Supplementary Fig. 4). We also tested if genome GC content was a driver of variation in predicted lifespan and if it should be adjusted for within the model. However, there was no correlation between GC content and the absolute error rate (Supplementary Fig. 5). This analysis suggests the longevity model is independent of technical factors and variations within genomes. We also tested the lifespan clock on non-vertebrates using the raw prediction values (Supplementary Table 3 and Supplementary Text). However, the lifespan clock returned inaccurate estimates for non-vertebrates suggesting it is only suitable for vertebrate species.

We characterised the functions of the lifespan-related loci by performing a gene ontology (GO) enrichment with the associated genes detailed in EPD. Previous research has described an association between energy metabolism and lifespan^41,42^, often referred to as the rate-of-living theory^43,44^. However, although lifespan loci-associated genes were most commonly related to development and energy metabolism processes, there was no significant enrichment for any GO terms. We also performed Pearson correlations between lifespan and CpG density to determine which promoters positively and negatively correlated (Supplementary Table 2). Of the 42 promoters 34 correlated significantly (p < 0.05) with lifespan, of which 12 and 22 promoters correlated negatively and positively with lifespan respectively. The remaining 8 lifespan loci did not significantly correlate with lifespan.

### Extinct animal lifespan estimation

Lifespan is a central life-history attribute, so a lifespan estimator coupled with ancient DNA analysis can reveal this previously hidden aspect of the ecology of extinct species. We estimated lifespan for two extinct members of the Elephantidae family, the woolly mammoth (*Mammuthus primigenius*)^45^ and the straight-tusked elephant (*Palaeoloxodon antiquus*)^46^. By identifying single nucleotide polymorphisms (SNPs) into the African elephant genome we were able to estimate lifespan estimates for these two extinct species. The AnAge database lists the African elephant as having an estimated lifespan of 65 years, which was used in training data set. The lifespan clock estimated both the woolly mammoth and the straight-tusked elephant as having a lifespan of 60.0 years. Although this is within range of the modern-day counterpart due to the lack of lifespan information surrounding the woolly mammoth and the straight-tusked elephant, it is difficult to determine the true accuracy of the model for these two species. There is no *a priori* reason that accuracy of estimates of lifespan for extinct species should be less than living ones (median 1.2%, 3.72 years in the testing dataset). We also analysed the passenger pigeon (*Ectopistes migratorius*) which has an assembled genome^47^ and became extinct in 1914^48^. The lifespan clock estimated the lifespan for the passenger pigeon to be 28.0 years. The lifespan of the passenger pigeon in the wild was never recorded. However it has been suggested that the age of Martha, the last surviving member was at least 17 and more likely, as old as 29 years^49,50^, which, although only a single example, adds credibility to our model-based estimate of lifespan.

We also examined whether lifespan estimates for humans significantly differed from their close relatives, including chimpanzees^51,52^ and extinct members of the Hominidae family, Denisovans^53^ (*Homo denisova*) and Neanderthals^54^ (*Homo neanderthalensis*). The lifespan clock estimated a 38.0 year lifespan for humans (hg19). The maximum lifespans of humans is a controversial topic^55,56^. In the past 200 years, the average life expectancy of humans has more than doubled because of modern medicine and changes in lifestyle^57,58^. Early humans have been reported to have a maximum life expectancy of 40 years^57,58^ less than half by modern standards^23,38^. Similarly, in chimpanzees the lifespan was estimated at 39.7 years. The maximum longevity of a chimpanzee in the wild is thought to be of a 55 years old female, however it is reported that many live to approximately 40 years of age^23,59^. We next estimated the lifespan of Denisovans and Neanderthals. We estimated that Denisovans and Neanderthals both had a lifespan of 37.8 years. This suggests that these extinct Hominidae species had similar lifespans to their early human modern-day counterparts.

### Lifespan estimation in long-lived species

The Rougheye rockfish (*Sebastes aleutianus*) was the oldest lived species in the data set at 205 years. Some species of tortoises and whales have also been reported to live for more than 100 years^60,61^. These species are of interest as they can provide models and insights into longevity and age associated diseases, but they are also difficult species for which to obtain lifespan estimates. We explored the application of the lifespan clock to several very long-lived species which were not included in the training data set. We first tested the lifespan clock on the genome of the Pinta Island tortoise (*Chelonoidis abingdonii*), which has a lifespan within the calibration range^62^. Lonesome George was the last surviving Pinta Island tortoise and was estimated to be over 100 years old when his genome was sequenced. The lifespan clock estimated the maximum lifespan of the Pinta Island tortoise to be 120 years old. This lifespan estimation is 10–20 years higher than most estimates of Lonesome George’s age at death^62^. It is important to note that this is not the accepted maximum lifespan of Pinta Island tortoise due to only one individual having its age recorded at death. Nevertheless, the model provides a credible and rigorously validated lifespan estimate for this long-lived and extremely data deficient species. Application of the model to other species of Galapagos tortoise with better lifespan information would enable further evaluation of the lifespan estimate for *Chelonoidis abingdonii*.

Bowhead whales are thought to be the longest living mammal^19^, with one individual estimated as 211 years old^19^. Using our lifespan estimator and the bowhead whale genome^63^, we estimated the maximum longevity of the bowhead whale to be 268 years. This lifespan estimate is 57 years more than the oldest aged individual to date^19,63^. Lifespan estimation for long-lived species is difficult since many age estimates have been made by extrapolation with models calibrated on limited data from much younger known-age individuals. Bowhead whales provide another example of this, with lifespan predicted by the alternative method of eyeball amino acid racemisation^19^ being well beyond the calibration range of the model, as it is with our lifespan clock. Moreover, it is rarely possible to follow long-lived species from birth to death as they would normally out live a generation of researchers. It is also important to note that many of the age estimates in these animals showed no signs of pathology^19^. Generally, if an animal was in the upper limits of its lifespan one would expect pathological features of some age-related diseases. The lack of such findings suggest that the animals were not near the maximum of their lifespans and may potentially had lived for many years longer.

## Discussion

By analysing CpG density in genomes of vertebrates with known lifespans we have identified 42 gene promoters that can be used as highly effective biomarkers of lifespan. This is novel research, as previous study have investigated associations but not using CpG density as predictive biomarker^33^. We were able to test our lifespan clock on the five most speciose classes of vertebrates, demonstrating the potential to predict lifespan from CpG density. We were unable to accurately estimate the lifespans of non-vertebrates, possibly many invertebrates do not exhibit DNA methylation to the extent as vertebrates^64–66^. Moreover, we found the CpG density around the transcription start site to have a distinct pattern in insects compared to vertebrates (Supplementary Text), similar to what has been shown in a previous study^67^. Nevertheless, we were able to apply the lifespan clock to long-lived vertebrate species, and other groups for which it is difficult to obtain lifespan estimates. However, any genetic regulation for a species may potentially be a secondary factor as there may be other environmental selective pressures. This may be the case with species which have lifespans post reproductive age and therefore, there may be non-genetic factors that may be more predictive of their maximum lifespan.

It has been suggested that DNAm patterns are not maintained with aging and that CpG density may be associated with gene expression relating to increasing longevity^33^. CpG density is associated with lifespan in mammals^33^. However, this study has shown that this also occurs in other vertebrates. Despite promoters being relatively evolutionary conserved^34^, CpG sites are prone to mutations^68^ which may make them targets for selection, including changes in lifespan. There may be the potential for CpG density to be under selective pressure as it may be regulating the expression of genes involved in longevity. Furthermore, the poor performance of our model in non-vertebrates may reflect an alternative function and genomic distribution of CpG sites in non-vertebrates^69^.

Lifespan is a significant parameter in population biology, and the lifespan clock therefore has diverse potential applications in the study and management of wild animal populations. For example, for modelling the fate of endangered species through population viability analyses (PVA)^70^. In the case of fisheries, harvest is often managed by setting catch limits based on population models incorporating natural mortality rate (*M*). However, *M* is one of the most difficult population traits to estimate for fish^71,72^. The lifespan clock has a clear application for life history-based *M* estimation because lifespan and *M* are strongly correlated and *M* can be estimated with little extra information once lifespan is known^73^. A key advantage of lifespan-based *M* estimators over alternative methods is that they provide a rapid estimate, avoiding inappropriate harvest of newly exploited stocks that in the past has resulted in major fisheries collapse such as the case with the orange roughy (*Hoplostethus atlanticus*)^74^. The capacity to generate *M* estimates from genome-based lifespan estimates will be particularly valuable for assessment of recent, lower trophic level fisheries that generally have poorly understood population biology^75^.

Despite the high predictive value and potential importance of our model for a broad variety of wildlife applications, there are several caveats that must be considered when interpreting its results. Firstly, the model relied on lifespan estimates from the AnAge database. Although AnAge is the world’s largest lifespan database and undertakes its own quality assessment, it does contain a mix of estimates from animals in captivity and in the wild^76^. Therefore, the lifespan estimated for species are likely over-estimates of what would normally be reached within the wild. Second, due to the limited number of species with both genomes and lifespan estimates we chose to use all available species in a combined analysis. This provides a universal lifespan predictor for vertebrates, but our results also suggest that there may be taxon-specific relationships between CpG density and lifespan. All vertebrate classes had statistically significant regression coefficients with the known and predicted lifespans but with varying correlations. Not surprisingly, mammals had the strongest correlation, but this may be due to the model being developed with human specific promoters from EPD. Mammals are the most evolutionary recent vertebrate class^77^ and therefore promoter sequences have had less divergence time compared to other classes. It would therefore be ideal in future research to recalibrate the model specific to taxonomic classes using class specific promoter databases once more data becomes available. In the future class-specific models may provide greater predictive power but will require the availability of significantly more genomes to be practical. Nevertheless, despite these limitations, the lifespan clock provides a remarkable level of predictive power across a very diverse group of organisms.

## Conclusions

In this study, we have shown that CpG density correlates strongly with lifespan across the five most speciose vertebrate classes. Our results also enabled the construction of a model that can predict lifespan accurately from only a small number of genomics features. The lifespan clock has broad applications to questions about the population biology of extant and extinct species. Lifespan is a central component of models for managing harvested wild species such as fish, sharks and threatened wild species, where lifespan is critical in determining sustainable harvests and population viability. The lifespan clock also creates a new opportunity to study the biology of extinct species, where ancient DNA methods can provide genome assemblies. Our study adds lifespan to the range of significant ecological parameters that can be provided by molecular biology.

## Methods

### Promoter sequence conservation and CpG density calculation

We used promoter sequences centred around the transcription start site (TSS) (-499 to 100 bp of each promoter) in Humans (*Homo sapiens*) from the EPD^36^ as the data set of promoter sequences. We chose the human data set as it has the largest number of promoter sequences (29,598 promoters) compared to other species available and has been experimentally verified using a range of high-throughput transcription start site mapping methods^36^. Briefly, as described previously^33^, using Basic Local Alignment Search Tool (BLAST) v2.2.31 the promoter sequences were mapped to the single top hit in each species. A significant hit was considered in a species with an identity >70%. CpG density was determined by counting the total number of CpG sites in the promoter for each species and dividing it by the BLAST hit length. Where a hit was not identified for a promoter in a given species, the CpG density was considered to be 0.

### Lifespan prediction modelling

To estimate lifespan from CpG density we used maximum lifespan data available from AnAge^23^. The AnAge database contains lifespan estimates of animals from a wide variety of sources including animals kept in captivity and in the wild. Despite AnAge containing other phenotypic data including various body traits, pregnancy related information such as gestation length and clutch size, which may have associations with lifespan this data was excluded from any lifespan prediction modelling. This was due to the data being too sparse to be advantageous to be included within the modelling. In addition, it also makes the model independent of such factors which may be unobtainable or unknown for many species.

In total, 252 species (Supplementary Table 1), with the exclusion of humans, contained reference genomes, available from NCBI genomes (https://www.ncbi.nlm.nih.gov/genome/),maximum lifespan data in AnAge and evolutionary divergence times in TimeTree. The AnAge database is a meta-analysis of other studies that have reported lifespan of species within the literature. Of the 252 species used in this study from the AnAge database, 151 were from animals kept in captivity, 84 in the wild and 17 from undetermined captive or wild sources. In addition, the lifespan values from AnAge of the 252 species were from multiple sources and had large (>1000) sample sizes. The taxonomic classification of animals was kept as detailed on NCBI. Known lifespans were natural log transformed to enable the data to fit a linear model. Species were randomly assigned to either a training (176 samples) or testing (76 samples) data set (70/30 split). An equal representation of each taxonomic class was maintained between the training and testing data by using the createDataPartition function within the caret R package^78^. The average divergence times was determined for both the training (mean = 575 MYA) and testing (mean = 584 MYA) data sets to determine if there was a bias of closely related species being in one of the data sets. However, we found no significant difference (p = 0.6391, t-test), suggesting that there is no overrepresentation of closely related species in either the training or testing data set. The training data set was subject to an elastic net penalized regression model^79^ where the lifespan of the species was used to regress against the CpG density of the 29,598 promoters. The glmnet function was set to a 10-fold cross validation which returns the best performing model. The α-parameter of glmnet was 0.5 and the minimum λ-value based on the training data was 0.16539. This resulted in a total of 42 promoters for estimate lifespan. The model returns the most informative promoters, however it does allow some redundancy to increase robustness^79^. A PGLS was performed using downloaded divergence times from TimeTree^80^ and the caper and APE^81^ packages in R. Vertebrate classes were also incorporated into the model resulting in a specific model for each specific class. PGLS was trained on the raw prediction values (sum of the product of coefficient weights multiplied by the respective CpG densities and coefficient intercept) (Supplementary Table 2). The final formula to estimate lifespan is:

$$ln\,(maximum\,lifespan)=-\,4.38996+2.57328x+ax+b$$

where *x* is the raw summed CpG density weight per sample, *a* and *b* are coefficients dependent on vertebrate class Table 1). The testing data set was used to validate the model. Correlations between the known and estimated lifespans and the MAE were determined to assess the performance of the model.

**Table 1:** Coefficients to estimate the lifespan of animals in specific vertebrate classes.

Class	*a*	*b*
Aves	−0.90323	2.14857
Fish	2.14632	−6.58228
Mammalia	−0.92888	2.33508
Reptilia	−0.48958	1.17281

### Principle component analysis

A PCA was used to determine which characteristics separate the species using the CpG density in the lifespan loci. This would also provide insight into other variables which may need to be accounted for within the model. The PCA was performed using the PCA function in FactoMineR^82^.

### Ancient genome lifespan estimation

To determine the lifespan of ancient genomes for species that have become extinct we used BAM files that had been mapped to the closest relative with a reference genome. Samtools v1.3 and bcftools v1.6 were used to identify single nucleotide polymorphisms (SNPs) within the BAM files of the extinct species compared to the relevant reference genome. SNPs within the lifespan loci have the potential of gaining or losing CpG sites, thereby altering CpG density. Using the locations of the lifespan loci in the relevant reference genomes, CpG density was calculated as described above. BAM files available from the European Nucleotide Archive (ENA) for the woolly mammoth (SAMEA3340290)^45^ and straight-tusked elephant (SAMEA24850918), along with the African elephant genome LoxAfr 3.0 and LoxAfr 4.0 respectively, as these were the versions of the genomes used in each study, were used for lifespan estimation. For Denisovans and Neanderthals lifespan estimation, we downloaded BAM files from the original studies^53,54^ which had been mapped to the human genome (hg19).

### CpG density surrounding the transcription start sites

To determine the CpG density around the TSS for each species, we used the fasta and gff files from NCBI genomes. The 5kbp upstream and downstream sequences of each TSS was divided up into 500 bp bins using bedtools v2.25.0^83^. The CpG density was determined by counting the total number of CpG sites within each bin. The function NbClust^84^ was used to determine the total number of CpG clusters in the data. This function provides 30 indices including the gap statistic and silhouette method and determines the total number of clusters by majority rule. CpG density distributions between species were tested for statistically significant differences using a Kolmogorov–Smirnov test implemented in R.

### Gene ontology and evolutionary distance

Gene ontology (GO) enrichment was performed using Enrichr^85^ using the 2018 terms. GO terms were considered significant if the adjusted p-value < 0.05. All analyses within R were performed using R version 3.5.1. Evolutionary distance was determined using TimeTree^37^ which uses multiple sources from the literature to determine the median time when species diverged.

## Supplementary information

Supplementary Information

Supplementary Table 1

Supplementary Table 2

Supplementary Table 3
