# Supplementary material for: A genomic predictor of lifespan in vertebrates
Source: Sci Rep. 2019 Dec 12;9:17866. doi: 10.1038/s41598-019-54447-w (PMC6908713; doi:10.1038/s41598-019-54447-w)
Supplement: Supplementary file 1 — Supplementary Information [file 41598_2019_54447_MOESM1_ESM.docx]

A genomic predictor of lifespan in vertebrates

Benjamin Mayne^1,*^, Oliver Berry^1^, Campbell Davies^2^, Jessica Farley^2^ and Simon Jarman^1,3^

^1^ Environomics Future Science Platform, Indian Oceans Marine Research Centre, Commonwealth Scientific and Industrial Research Organization, Crawley, Western Australia, Australia

^2^ Oceans and Atmosphere, Commonwealth Scientific and Industrial Research Organization, Hobart, Tasmania, Australia

^3^ School of Biological Sciences, University of Western Australia, 35 Stirling Highway, Perth, Western Australia, Australia

* Author correspondence: [benjamin.mayne@csiro.au](mailto:benjamin.mayne@csiro.au)

**Lifespan estimation in non-vertebrates**

CpG islands associated with promoters also occur in non-vertebrates. The transcriptional regulation function of these CpG islands are thought to differ somewhat to that observed in vertebrates, but we were interested in exploring whether our lifespan clock had predictive power outside vertebrates ^1^. Few non-vertebrates have reported maximum lifespans ^2^ and were therefore excluded from the lifespan clock model. In this analysis we compared the lifespan estimates to sources within the literature. We tested our lifespan estimator on all non-vertebrates (Supplementary Table 4) with reference genomes in NCBI. The lifespan clock over-estimated the lifespan for many species. For example, the common fruit fly (*Drosophila melanogaster*) has a reported lifespan of up to 60 days under optimal conditions ^3^. The lifespan clock predicted a mean lifespan across 37 species of *Drosophila* of 16.3 years (Supplementary Table 3), which is a gross over estimate ^3-5^. Although the lifespan of many insects is within the order of days ^6^, the lifespan of the reproductive females (the queens of the colony) can be up to 500 times longer than the short-lived males ^7^. This is the case with social insects such as ants, termites, bee and wasp species where the queen can live up to 30 years of age ^7^. However, the lifespan estimates were approximately half of reported queen lifespans and were therefore not reflective of longer lived individual insects.

The inaccuracy of the lifespan clock in insects may have result from known differences in CpG patterns between insects and vertebrates. For example, vertebrates generally exhibit genome-wide methylation whereas in many non-vertebrates, such as insects, methylation primarily occurs within coding sequences ^1,8^. To determine if CpG patterns in insects differed from vertebrates we characterised the CpG density around the transcription start site (TSS) (Supplementary Figure 6). The five vertebrate classes had similar patterns (Supplementary Figure 6), however insects had a significantly higher CpG density around the TSS than other taxa (Supplementary Table 4). This is consistent with previous observations ^1^ and may contribute to the over-estimated lifespan estimation in insects. This analysis suggests the lifespan clock is restricted to application in vertebrates because the underlying molecular biology of the process it measures is not conserved outside this group.

**References**

1 Glastad, K. M., Hunt, B. G., Yi, S. V. & Goodisman, M. A. D. DNA methylation in insects: on the brink of the epigenomic era. *Insect Molecular Biology* **20**, 553-565, doi:doi:10.1111/j.1365-2583.2011.01092.x (2011).

2 Tacutu, R. *et al.* Human Ageing Genomic Resources: new and updated databases. *Nucleic acids research* **46**, D1083-d1090, doi:10.1093/nar/gkx1042 (2018).

3 Linford, N. J., Bilgir, C., Ro, J. & Pletcher, S. D. Measurement of lifespan in Drosophila melanogaster. *Journal of visualized experiments : JoVE*, 50068, doi:10.3791/50068 (2013).

4 Fernández-Moreno, M. A., Farr, C. L., Kaguni, L. S. & Garesse, R. Drosophila melanogaster as a model system to study mitochondrial biology. *Methods in molecular biology (Clifton, N.J.)* **372**, 33-49, doi:10.1007/978-1-59745-365-3_3 (2007).

5 Sun, Y. *et al.* Aging studies in Drosophila melanogaster. *Methods in molecular biology (Clifton, N.J.)* **1048**, 77-93, doi:10.1007/978-1-62703-556-9_7 (2013).

6 Johnson, C. A. *et al.* Effects of temperature and resource variation on insect population dynamics: the bordered plant bug as a case study. *Functional ecology* **30**, 1122-1131, doi:10.1111/1365-2435.12583 (2016).

7 Jemielity, S., Chapuisat, M., Parker, J. D. & Keller, L. Long live the queen: studying aging in social insects. *Age (Dordrecht, Netherlands)* **27**, 241-248, doi:10.1007/s11357-005-2916-z (2005).

8 Jeong, H., Wu, X., Smith, B. & Yi, S. V. Genomic Landscape of Methylation Islands in Hymenopteran Insects. *Genome biology and evolution* **10**, 2766-2776, doi:10.1093/gbe/evy203 (2018).

**Supplementary Table 4.** p-values detailing the CpG density significance between different classes of species.

|  | **Amphibia** | **Aves** | **Fish** | **Insects** | **Mammalia** | **Reptilia** |
| --- | --- | --- | --- | --- | --- | --- |
| **Amphibia** |  |  |  |  |  |  |
| **Aves** | 1.43E-07 |  |  |  |  |  |
| **Fish** | 1.13E-08 | 0.00027 |  |  |  |  |
| **Insect** | 1.45E-11 | 1.45E-11 | 1.45E-11 |  |  |  |
| **Mammalia** | 1.43E-07 | 0.174533 | 0.001116 | 5.80E-10 |  |  |
| **Reptilia** | 9.55E-06 | 1.33E-06 | 1.43E-07 | 1.45E-11 | 9.55E-06 |  |


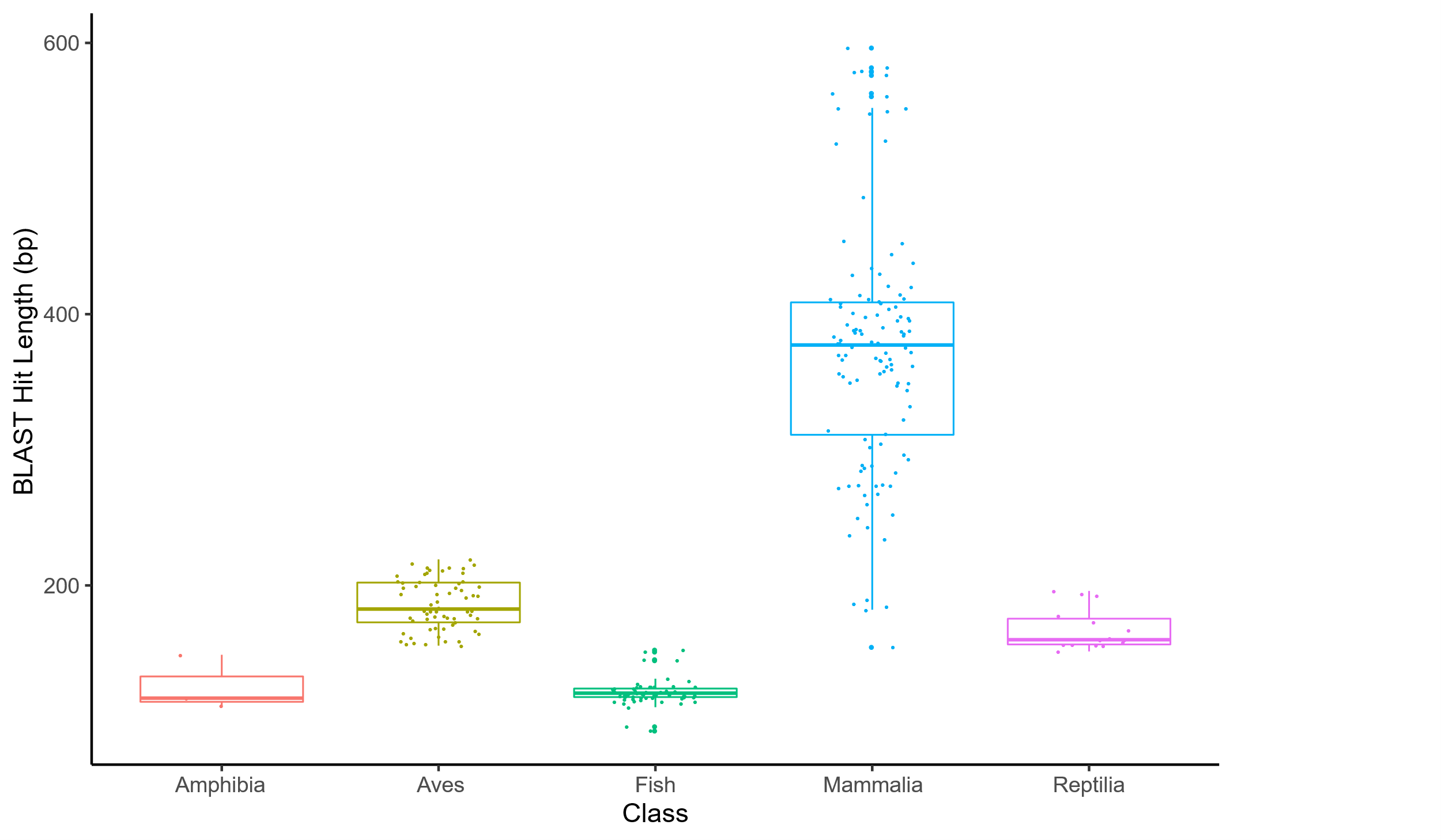


**Supplementary Figure 1:** Mean BLAST hit lengths for each species grouped by class. Each dot represents an individual species.


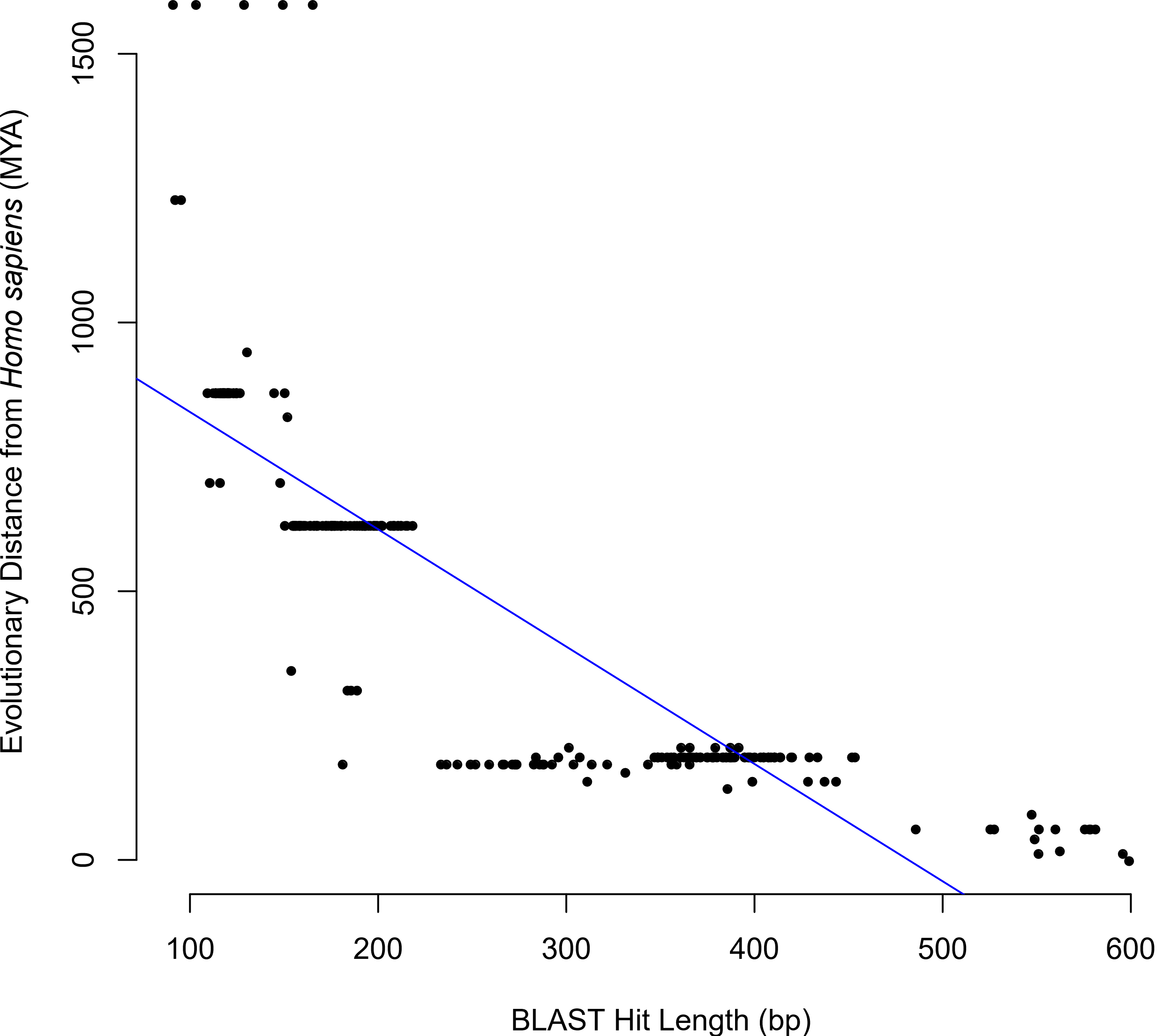


**Supplementary Figure 2:** Correlation between the BLAST hit length and the divergence time from humans for each individual species. Each dot represents an individual species and the divergence time is in the order of Millions of Years Ago (MYA). The blue line shows the correlation between the BLAST hit and divergence time from humans.


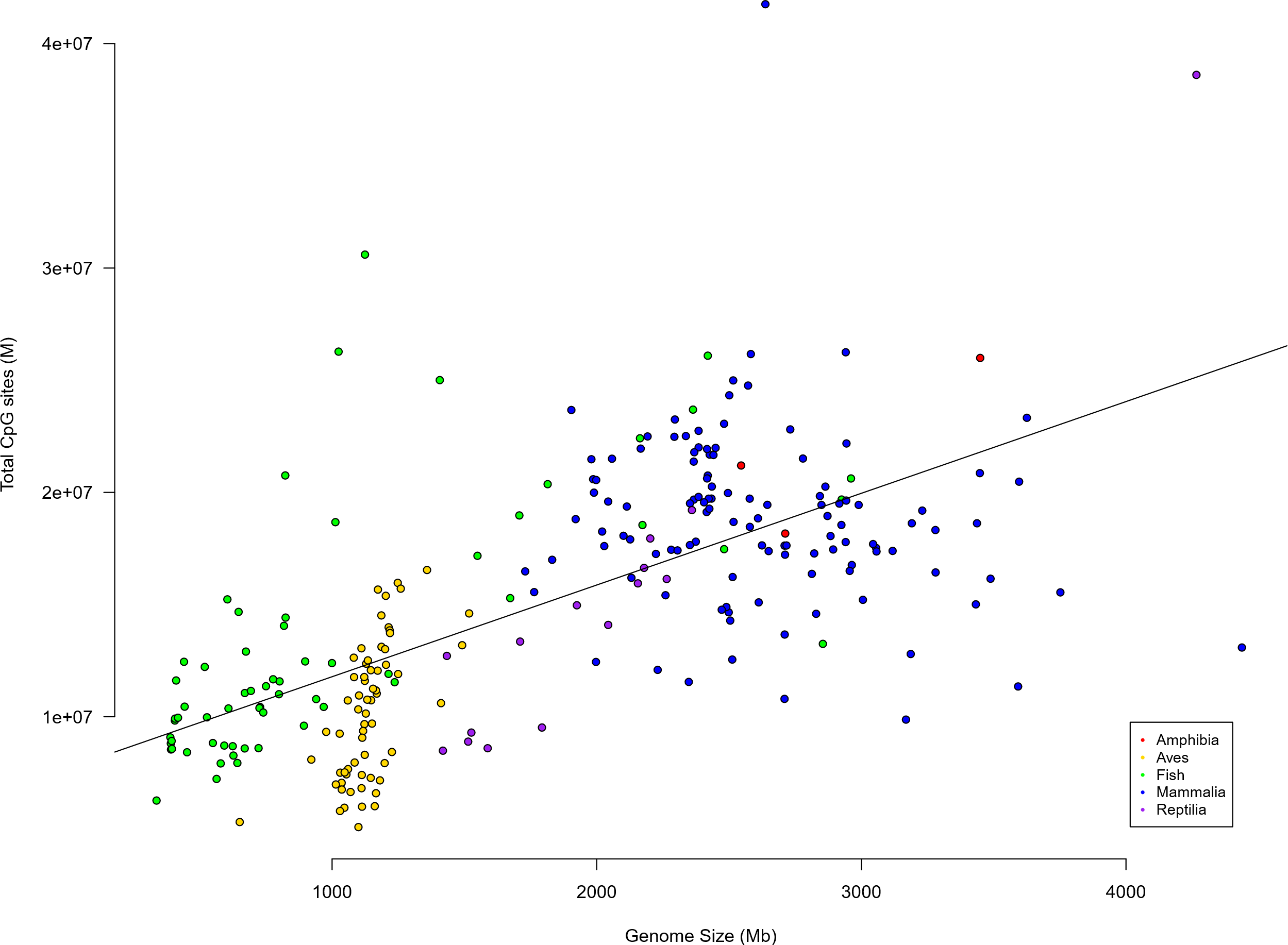


**Supplementary Figure 3:** Correlation between the genome size for each class of species and the average total number of CpG sites. Genome size is in mega-bases (Mb) and the total CpG sites is in the order of Millions (M).


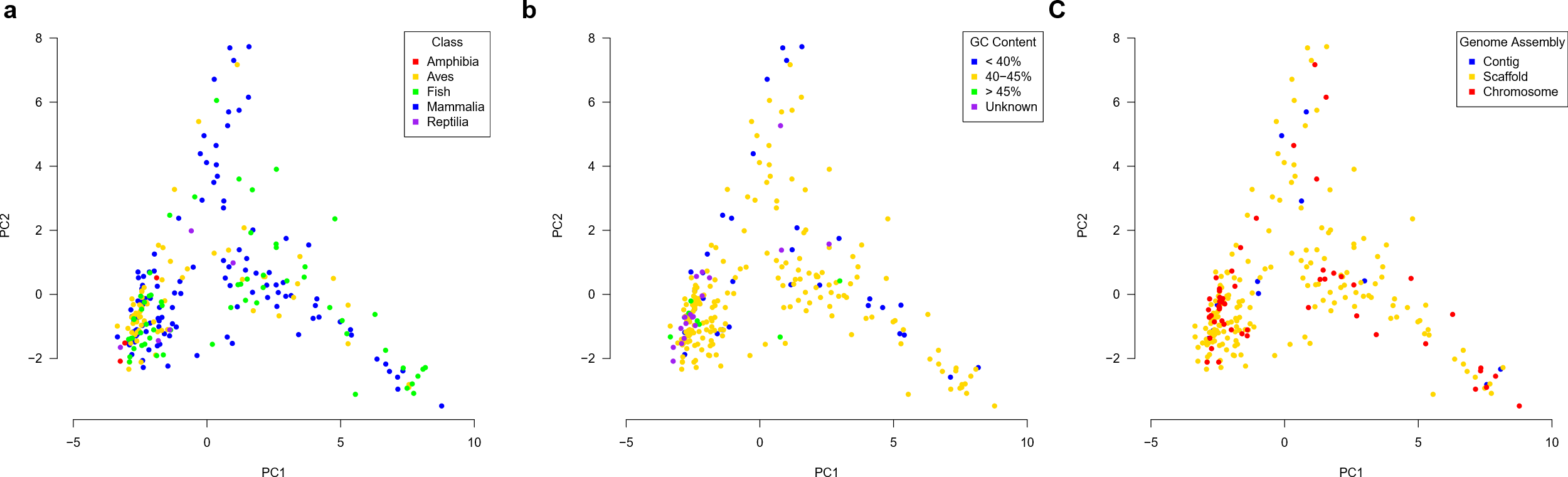


**Supplementary Figure 4:** Principle component analysis of lifespan loci coloured by groups showing any potential clustering of genomic or technical variations. Species’ do not cluster by **a,** class, **b,** GC content or **c,** level of genome build.


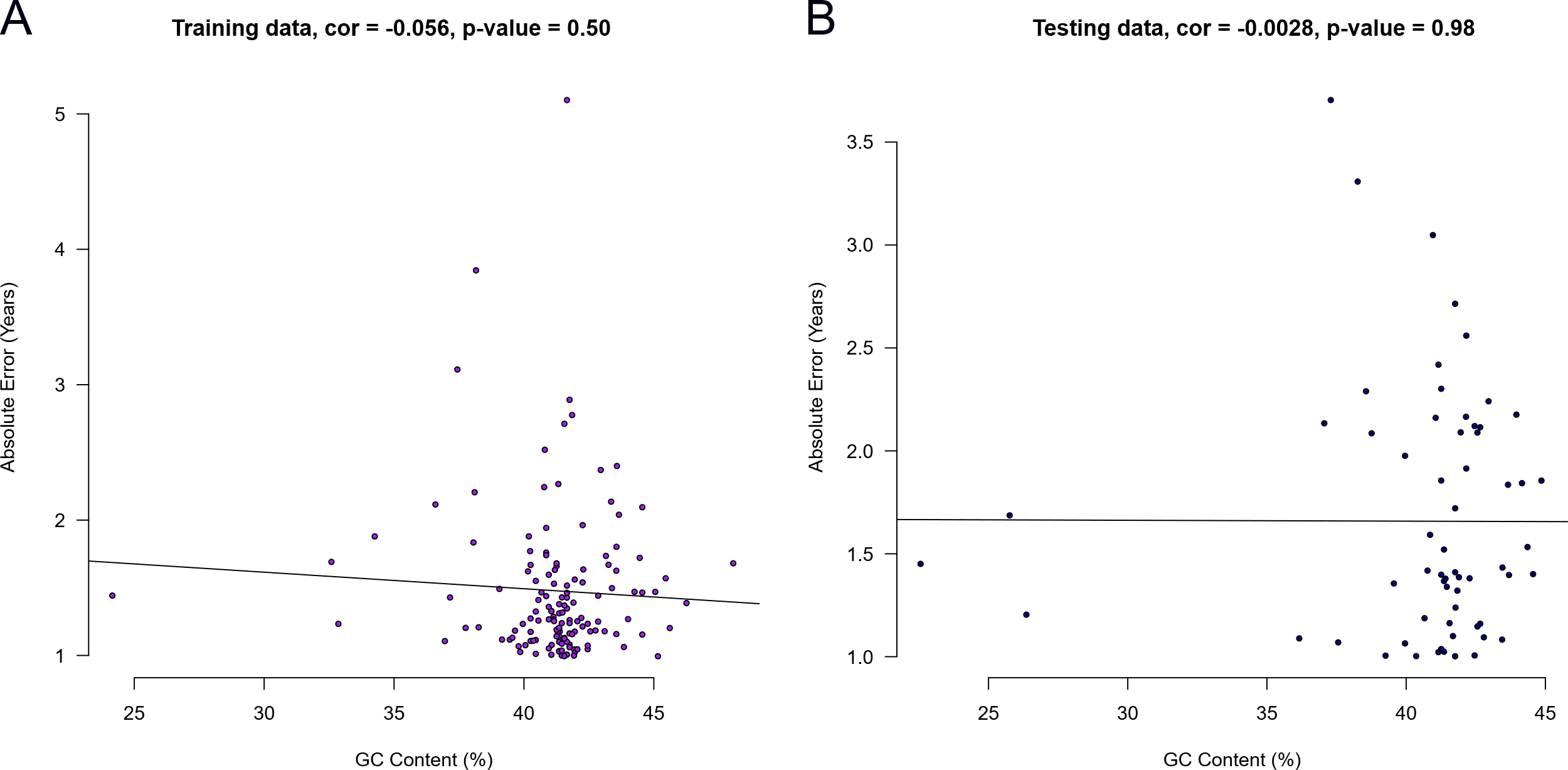


**Supplementary Figure 5:** Correlation between GC content (%) and the absolute error rate per species in **a**, the training data and **b**, the testing data.


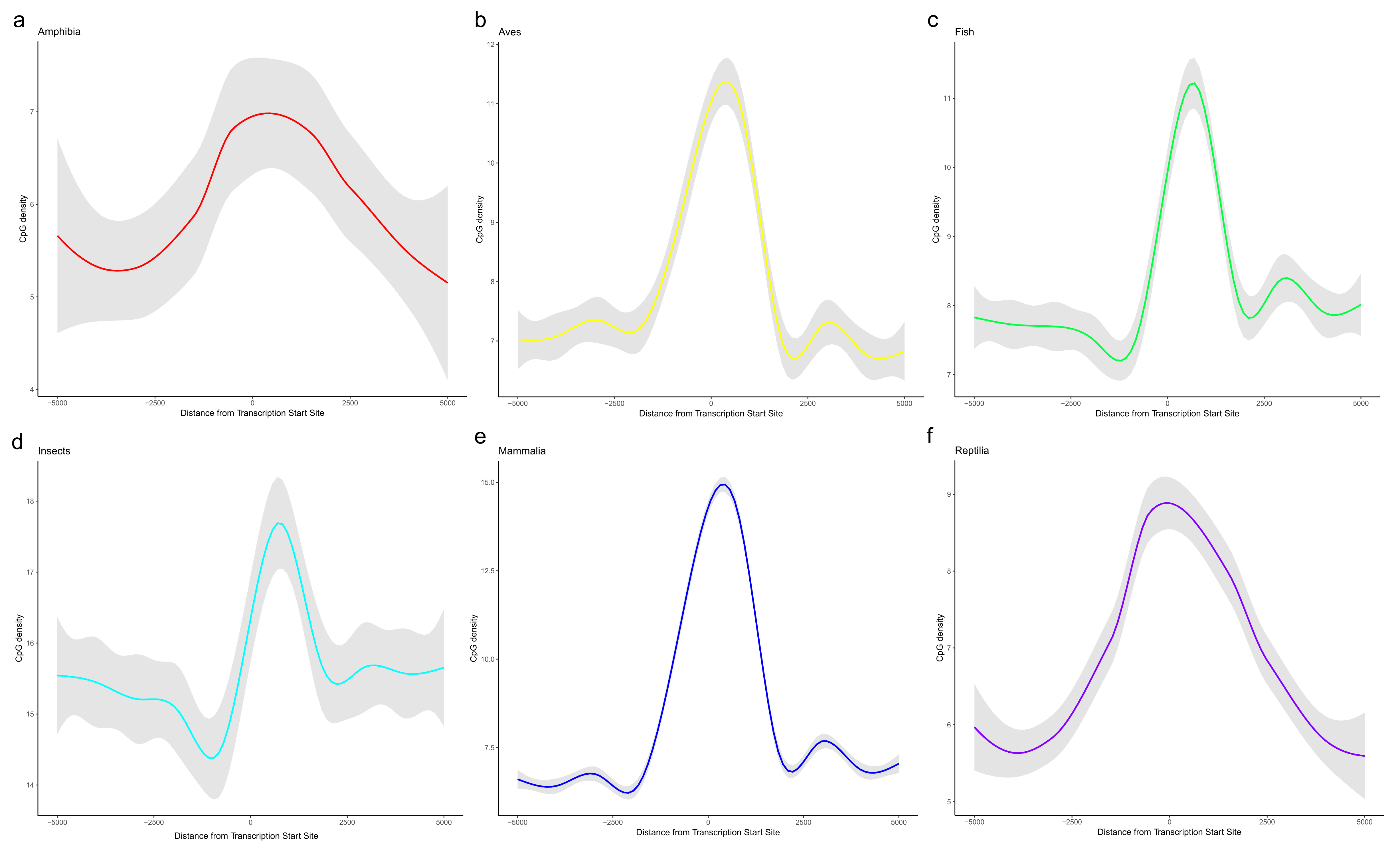


**Supplementary Figure 6:** CpG density around the transcription start site between different classes. Shaded grey areas represent the standard deviation of CpG density and coloured lines show the mean trace.
